# Bioelectrical impedance analysis-derived phase angle as a determinant of protein-energy wasting and frailty in maintenance hemodialysis patients: retrospective cohort study

**DOI:** 10.1186/s12882-020-02102-2

**Published:** 2020-10-19

**Authors:** Masakazu Saitoh, Masumi Ogawa, Hisae Kondo, Kiichi Suga, Tetsuya Takahashi, Haruki Itoh, Yoichiro Tabata

**Affiliations:** 1 Department of Physical Therapy, Faculty of Health Science, Juntendo University, 3-2-12, Hongo, Bunkyo-ku, Tokyo, 113-0033 Japan; 2 Department of Rehabilitation, Sakakibara Heart Institute, Tokyo, Japan; 3 Meiseikai Toyo Clinic Yachimata, Chiba, Japan; 4 Department of Cardiology, Sakakibara Heart Institute, Tokyo, Japan

**Keywords:** Bioimpedance electrical analysis, Frailty, Hemodialysis, Phase angle, Protein energy wasting

## Abstract

**Background:**

Phase angle (PA), measured by bioelectrical impedance analysis (BIA) has been studied as indicator of nutritional status or muscle function in hemodialysis (HD) patients. It remains unclear if the phase angle is associated protein-energy wasting (PEW) or frailty, which are common complication in hemodialysis patients. The aim of this study is to determine whether BIA-derived PA is a marker of PEW or frailty in HD patients.

**Methods:**

This retrospective observational study included 116 adult HD patients (35% female, 64 ± 12 years of age) in a single dialysis center. Patients were classified according to the PA quartiles into four groups; 1) first quartile: PA < 3.7°, 2) second quartile: PA 3.7–4.1°, 3) third quartile: PA 4.2–4.9°and 4) forth quartile: PA ≥ 5.0°. International Society of Renal Nutrition and Metabolism (ISRNM) criteria and Japanese version of Cardiovascular Health Study (J-CHS) criteria were used to identify PEW and frailty.

**Results:**

The lower PA group was associated with a greater risk of PEW (35% vs. 24% vs. 21% vs. 3%; *p* = 0.032), frailty (59% vs. 40% vs. 21% vs. 3%; *p* < 0.001). In multivariate logistic regression analysis, the first quartile group was at a significantly greater risk of both PEW and frailty compared with the fourth quartile group after adjusting for other confounding factors.

**Conclusions:**

Lower PA was associated with a greater risk of PEW and frailty in HD patients.

## Background

Protein-energy wasting (PEW), defined as the loss of somatic and circulation body protein and energy reserves, is a common complication among hemodialysis (HD) patients [1]. Frailty can be defined as a biological syndrome of decreased reserve and resistance to stressors that results from cumulative decline across multiple physiological systems and is highly prevalent in patients with HD. HD patients accompanied by PEW or frailty are associated with accelerated biological ageing, and an increased risk of cardiovascular event (CV) and death [1, 2]. A more recent proposal suggests early screening and diagnosis of PEW and frailty are important in clinical practice among HD patients [1–3].

Bioimpedance electrical analysis (BIA) is a noninvasive, safe and quick methods, and validated assessment of hydration status and body composition. The clinical use of BIA in HD patients is currently increasingly used in dry weight and nutritional status management. Phase angle (PA) measured by BIA reflects the resistance and reaction of the body in response to the application of an external current. PA is the most clinically relevant impedance parameter, an index of cell membrane integrity and vitality. Phase angle is a direct measure of BIA and therefore not influenced by assumptions that can affect body composition or hydration assessments. A lower PA level indicates decreased cell integrity or cell death, whereas higher PA suggests large quantities of intact cell membranes [4]. Moreover, PA has been recently used as a tool for assessing disease progression as well as for predicting clinical outcome in many clinical situations [5]. However, the association between PA and PEW or frailty remains uncertain among HD patients. This study focused on HD patients, primarily to investigate the validity of BIA-derived PA in predicting PEW and frailty, and secondly to examine the association between PA and CV risk.

## Methods

### Subjects

The present study included 116 adult HD patients (35% female, 64 ± 12 years of age) from a single unit of the Meisei-kai Toyo Clinic Yachimata, Chiba, Japan between January 2018 and March 2018. Patients were eligible to participate if they were over 18 years of age, had received maintenance HD at least 3 times per week for more than 6 months, and had no contraindications for BIA including patients with pacemakers or were not limbless. The exclusion criteria of present study were comorbidities active malignancy and recent hospital admission within 3 months that might influence nutritional or functional status. Study collaborators interviewed patients before or during a HD session, obtained recent clinical and laboratory data from medical records, and measured muscle strength and physical performance prior to the start of the HD session. In addition, study collaborators measured the body composition using a BIA after a midweek dialysis session.

### Cardiovascular risk score

Cardiovascular (CV) risk score was calculated using new risk model developed by Japan Dialysis Outcomes and Practice Patterns Study (J-DOPPS) [6]. This CV risk model had a more accurate dose-dependent association with observed CV events than the Framingham risk score among HD patients. The J-DOPPS CV risk model contained only six variables: age, diabetes mellitus, history of CV events, dialysis time per session, phosphorus level, and albumin level, ranging from 0 to 20 points with higher scores reflecting greater risk of CV events.

### Diagnosis of protein-energy wasting

International Society of Renal Nutrition and Metabolism (ISRNM) recommended the diagnosis of PEW [7]. PEW involves 4 categories: (1) serum chemistry: low serum albumin, or total cholesterol; (2) body mass: decreased body mass index (BMI) or total body fat percentage or unintentional weight loss, (3) muscle mass: pre-dialysis serum creatinine appearance normalized by the body surface area (sCr/BSA), and (4) dietary intake normalized protein nitrogen appearance (nPNA). The cutoff values were as follows: serum albumin, 3.8 g/dL; BMI, 23 kg/m^2^ or unintentional weight loss (5% over 3 months or 10% over 6 months); sCr/BSA, 380 μmol/L/m^2^; and nPNA, 0.8 g/kg per day. The diagnosis of PEW was defined at least 3 of the 4 listed categories.

### Diagnosis of frailty

Frailty was evaluated based on the Japanese version of Cardiovascular Health Study (J-CHS) criteria consisting of 5 components: weight loss, exhaustion, low physical activity, slowness and weakness [8]. (1) Weight loss was evaluated using the question “Have you lost 2kg or more in the past 6 months?”. (2) Exhaustion was measured using the question: “In the past 2 weeks, have you felt tired without a reason? ”. (3) Low physical activity was measured using the two questions: “Do you engage in moderate levels of physical exercise or sports aimed at health?”, and “Do you engage in low levels of physical exercise aimed at health?”. (4) Slowness was measured using usual gait speed: patients were asked to 5 m-walk at their comfortable pace using any walking aids to maintain balance and function. (5) Weakness was evaluated by measuring handgrip strength in the sitting position. The J-CHS comprises the following: (1) Weight loss: 1 point for “yes” to the question; (2) Exhaustion: 1 point for “yes” to the question; (3) Low physical activity: 1 point for “no” to both questions; (4) Slowness: 1point if gait speed < 1.0 m/s; (5) Weakness: 1point if handgrip strength < 26 kg in men and < 18 kg in women. Summing up the J-CHS scores, we calculated a total J-CHS score; a cut off of ≥3 was used to identify frailty.

### Bioelectrical impedance analysis

BIA measurements was performed by seca mBCA515 (seca®, Hamburg, Germany), which is a multifrequency bioimpedance device. The 8-electrode texhniqu enables segmental impedance measurement of the arm and legs. All the patients were in a standing position. The PA was calculated using the following equation:

PA(degree) = arctan(Xc/R) × (180/*π*)), is related to body cell mass and soft tissue composition.

Quartiles were obtained for the PA (25th, 3.7°; 50th, 4.2°; 75th, 5.0), and the patients were classified in four groups: first quartile group (PA < 3.7°), second quartile group (3.7 ≤ PA < 4.2°), third quartile group (4.2 ≤ PA < 5.0°), and fourth quartile group (PA ≥ 5.0°).

To examine the PA values adjusting for age, sex, and body mass index, the PA values were converted into s.d. score by the following equation:

Standard deviation score(SDS) = (X – average X)/s. d.

Where X is the observed value, average X is the mean of the normal value at the respective age, sex, and body mass index, and s.d. is the standard deviation from the mean. BIA-derived body components such as extracellular water (ECW) [9], total body water (TBW), fat mass and fat free mass were recorded, and ECW/TBW was calculated by the ratio of ECW and TBW.

### Statistical analysis

Continuous variables are expressed at mean ± standard deviation and as counts and percentages as appropriate. For the comparison of continuous variables among PA groups, one-way analysis of variance was used, and for categorical variables, the Pearson chi-square test was performed. To adjust for effects due to potential confounders for PA, multivariate logistic regression models of PEW, and frailty were performed, and odds ratios (ORs) and 95% confidence intervals (95% CI) were determined. The PA ≥ 5.0° (fourth quartile group) was considered the reference for this analysis. In the analysis for the CV event risk, we compared the CV event risk model score among PA groups using Kruskal-Wallis test. Statistical analyses were performed using SPSS software, version 21, and in all statistical calculations, a two-tailed *p* < 0.05 was considered statistically significant.

## Results

The average age of HD patients in the analysis was 64 ± 12 years; 35% of patients were female; dialysis vintage was 7 ± 6 years; PA score was 4.3 ± 1.1°, and PA SDS was − 1.1 ± 1.8; 65% of the patients had PA SDS < − 1 s.d., and 17% had PA SDS between − 1 and 0 s.d and 18% had PA SDS > 0 s.d..

The clinical characteristics of the study population according PA groups are shown in Table 1. HD patients with lower PA were significantly older, had a higher proportion of females, and lower BMI, serum creatinine level, albumin level, modified creatinine index, and handgrip strength. Our findings demonstrated that 35% of patients in the first quartile group, 24% in the second quartile group, 21% in the third quartile group, and 3% in the fourth quartile group exhibited PEW based on ISRNM criteria (*p* = 0.032). Moreover, the prevalence of frailty was 59% in the first quartile group, 40% in the second quartile group, 21% in the third quartile group, and 3% in the fourth quartile group (*p* < 0.001). The remaining clinical variables were not significantly different among PA groups. We evaluated the prediction accuracy of several variables measured by BIA. The area under the curve (AUC) value of a PA is large in predicting frailty and PEW compared with other values measured by BIA (Table 2).

**Table 1:** Clinical characteristics

	First quartile group (PA < 3.7) ***n*** = 29	Second quartile group (3.7 ≤ PA < 4.2) ***n*** = 29	Third quartile group (4.2 ≤ PA < 5.0) ***n*** = 29	Fourth quartile group (PA ≥ 5.0) ***n*** = 29	***P***-value among groups
Age, years	70 ± 10	67 ± 11	62 ± 11	58 ± 12	< 0.001
Female, n (%)	15 (52)	13 (43)	9 (31)	4 (14)	0.015
BMI, kg/m^2^	20.5 ± 3.2	24.6 ± 4.5	25.1 ± 5.6	24.6 ± 3.0	< 0.001
HD vintage, years	6.9 ± 7.6	7.1 ± 5.5	7.3 ± 4.3	6.0 ± 4.7	0.833
Hypertension, n (%)	28 (97)	29 (97)	27 (93)	27 (93)	0.864
Diabetes mellitus, n (%)	17 (59)	20 (67)	17 (59)	11 (38)	0.147
Hb, g/dL	10.2 ± 1.2	10.2 ± 1.0	10.7 ± 1.5	10.9 ± 0.9	0.054
sCr, mg/dL	8.8 ± 1.9	9.7 ± 2.1	11.6 ± 1.9	13.4 ± 2.4	< 0.001
Alb, mg/dL	3.6 ± 0.4	3.7 ± 0.2	3.7 ± 0.3	3.9 ± 0.3	0.005
P, pg/dL	5.2 ± 1.2	5.8 ± 1.2	5.6 ± 1.1	6.0 ± 1.6	0.104
Ca, mg/dL	8.6 ± 0.7	8.8 ± 0.8	9.0 ± 0.7	8.9 ± 0.6	0.129
K, mg/dL	5.1 ± 0.7	4.8 ± 0.8	4.9 ± 0.5	5.0 ± 0.7	0.308
CRP, mg/dL	0.58 ± 0.88	0.33 ± 0.44	0.26 ± 0.33	0.20 ± 0.20	0.045
Kt/V	1.4 ± 0.3	1.3 ± 0.2	1.3 ± 0.2	1.3 ± 0.2	0.667
nPNA, g/kg/day	0.8 ± 0.2	0.8 ± 0.1	0.8 ± 0.1	0.9 ± 0.2	0.088
EAT-10, points	1.6 ± 4.4	1.3 ± 2.9	0.6 ± 2.8	0.9 ± 2.8	0.702
SNAQ, points	14.4 ± 2.6	14.2 ± 3.2	15.0 ± 1.5	14.8 ± 1.6	0.563
Modified creatinine index, mg/kg/day	19.5 ± 1.9	20.5 ± 2.1	22.0 ± 2.8	24.3 ± 2.4	< 0.001
PA, °	3.0 ± 0.6	3.9 ± 0.2	4.7 ± 0.2	5.7 ± 0.5	< 0.001
PA SDS	−2.15 ± 1.60	−1.77 ± 1.17	−0.56 ± 1.07	1.08 ± 1.14	< 0.001
ECW/TBW	51.6 ± 0.7	48.0 ± 2.7	45.3 ± 2.0	41.4 ± 2.2	< 0.001
Handgrip strength, kg	17.8 ± 6.2	22.0 ± 7.4	26.3 ± 7.7	32.9 ± 7.6	< 0.001
SPPB score, points	10.3 ± 2.1	10.9 ± 1.7	11.0 ± 1.8	11.6 ± 0.9	0.055

*BMI* Body mass index, *HD* Hemodialysis, *Hb* Hemoglobin, *sCr* Serum creatinine, *BUN* Blood urine nitrogen, *Alb* Albumin, *P* Phosphorus, *Ca* Calcium, *K* Potassium, *CRP* C-reactive protein, *KT/V* K-dialyzer clearance of urea, *t* Dialysis time, *V* Volume of distribution of urea, *nPNA* Normalized protein nitrogen appearance, *SNAQ* Simplified Nutritional Appetite Questionnaire, *PA* Phase angle, *SDS* Standard deviation score, *ECT* Extracellular water, *TBW* Total body water, *SPPB* Short physical performance battery

**Table 2:** BIA measurements and protein energy-wasting, and frailty

	Frailty				Protein−energy wasting			
	AUC	SE	*p-*value	95% CI	AUC	SE	*p-*value	95% CI
Bioimpedance vector analysis R value	0.405	0.069	0.18	0.270–0.540	0.310	0.054	0.025	0.203–0.416
Bioimpedance vector analysis Xc value	0.291	0.062	0.003	0.169–0.413	0.362	0.078	0.104	0.211–0.514
PA	0.767	0.056	< 0.001	0.657–0.877	0.718	0.073	0.010	0.575–0.861
ECW value	0.577	0.069	0.279	0.442–0.712	0.603	0.067	0.222	0.473–7.340
TBW value	0.649	0.064	0.035	0.523–0.776	0.671	0.061	0.043	0.551–0.791
ECW by TBW value	0.286	0.059	0.002	0.171–0.401	0.324	0.067	0.038	0.194–0.455
Fat mass value	0.494	0.071	0.938	0.356–0.633	0.566	0.077	0.435	0.414–0.718
Fat free mass value	0.650	0.064	0.034	0.524–0.776	0.669	0.061	0.046	0.550–0.788

*PA* Phase angle, *ECW* Extracellular water, *TBW* Total body water

Table 3 shows the results of logistic regression analysis of the predictive variables related to PEW in HD patients. The univariate logistic regression analysis showed that the first quartile group (OR 14.737, 95%CI 1.740–124.827, *p* = 0.014) and second quartile group (OR 8.909, 95%CI 1.019–77.905, *p* = 0.048) were at significantly greater risk of PEW compared with fourth quartile group. Moreover, multivariate logistic regression analysis showed that first quartile group remained a predictor of PEW after adjusting for other confounding factors, compared to the fourth quartile group (model 2: OR 10.967, 95%CI 1.124–107.014, *p* = 0.039; model 3: OR 11.099, 95%CI 1.101–111.926, *p* = 0.041).

**Table 3:** Logistic regression analysis of the predictive variables related to PEW in hemodialysis patients

	Odds ratio	95% CI	***P***-value
Model 1			
Fourth quartile group (PA ≥ 5.0°)	1 (ref)		
Third quartile group (PA 4.2 to < 5.0°)	7.304	0.819–65.114	0.075
Second quartile group (PA 3.7 to < 4.2°)	8.909	1.019–77.905	0.048
First quartile group (PA < 3.7°)	14.737	1.740–124.827	0.014
Model 2			
Fourth quartile group (PA ≥ 5.0°)	1 (ref)		
Third quartile group (PA 4.2 to < 5.0°)	9.410	0.954–92.786	0.055
Second quartile group (PA 3.7 to < 4.2°)	8.294	0.847–81.215	0.069
First quartile group (PA < 3.7°)	10.967	1.124–107.014	0.039
Model 3			
Fourth quartile group (PA ≥ 5.0°)	1 (ref)		
Third quartile group (PA 4.2 to < 5.0°)	6.137	0.581–64.777	0.131
Second quartile group (PA 3.7 to < 4.2°)	7.607	0.757–76.454	0.085
First quartile group (PA < 3.7°)	11.099	1.101–111.926	0.041

Model 1: PA class

Model 2: PA class + age, sex, HD vintage

Model 3: PA class + age, sex, HD vintage, diabetes mellitus, hemoglobin, C-reactive protein

*PA* Phase angle, *HD* Hemodialysis

Table 4 shows the results of logistic regression analysis of the predictive variables associated with frailty in HD patients. In univariate logistic regression analysis, the first quartile group (OR 40.727, 95%CI 4.805–345.219, *p* = 0.001) and the second quartile group (OR17.111, 95%CI 2.031–144.136, *p* = 0.009) were at a significantly greater risk of frailty compared with the 4th quartile group. In multivariate analysis, the first quartile group (OR 36.770, 95%CI 3.906–346.140, *p* = 0.002) and the second quartile group (OR 16.525, 95%CI 1.867–146.285, *p* = 0.012) remained predictors of frailty after adjusting for age, sex, and HD vintage compared with fourth quartile group (model 2). Similarly, the first quartile group (OR 15.612, 95%CI 1.194–204.120, *p* = 0.036) was significantly associated with frailty after adjusting for age, sex, HD vintage, diabetes mellitus, hemoglobin level, grip strength than the fourth quartile group (model 3).

**Table 4:** Logistic regression analysis of the predictive variables related to frailty in hemodialysis patients

	Odds ratio	95% CI	***P***-value
Model 1			
Fourth quartile group (PA ≥ 5.0°)	1 (ref)		
Third quartile group (PA 4.2 to < 5.0°)	7.304	0.819–65.114	0.075
Second quartile group (PA 3.7 to < 4.2°)	17.111	2.031–144.136	0.009
First quartile group (PA < 3.7°)	40.727	4.805–345.219	0.001
Model 2			
Fourth quartile group (PA ≥ 5.0°)	1 (ref)		
Third quartile group (PA 4.2 to < 5.0°)	7.219	0.791–65.885	0.080
Second quartile group (PA 3.7 to < 4.2°)	16.525	1.867–146.285	0.012
First quartile group (PA < 3.7°)	36.770	3.906–346.140	0.002
Model 3			
Fourth quartile group (PA ≥ 5.0°)	1 (ref)		
Third quartile group (PA 4.2 to < 5.0°)	4.855	0.383–61.527	0.223
Second quartile group (PA 3.7 to < 4.2°)	9.315	0.803–108.108	0.074
First quartile group (PA < 3.7°)	15.612	1.194–204.120	0.036

Model 1: PA class

Model 2: PA class + age, sex, HD vintage

Model 3: PA class + age, sex, HD vintage, diabetes mellitus, hemoglobin, C-reactive protein

*PA* Phase angle, *HD* Hemodialysis

**Fig. 1:**
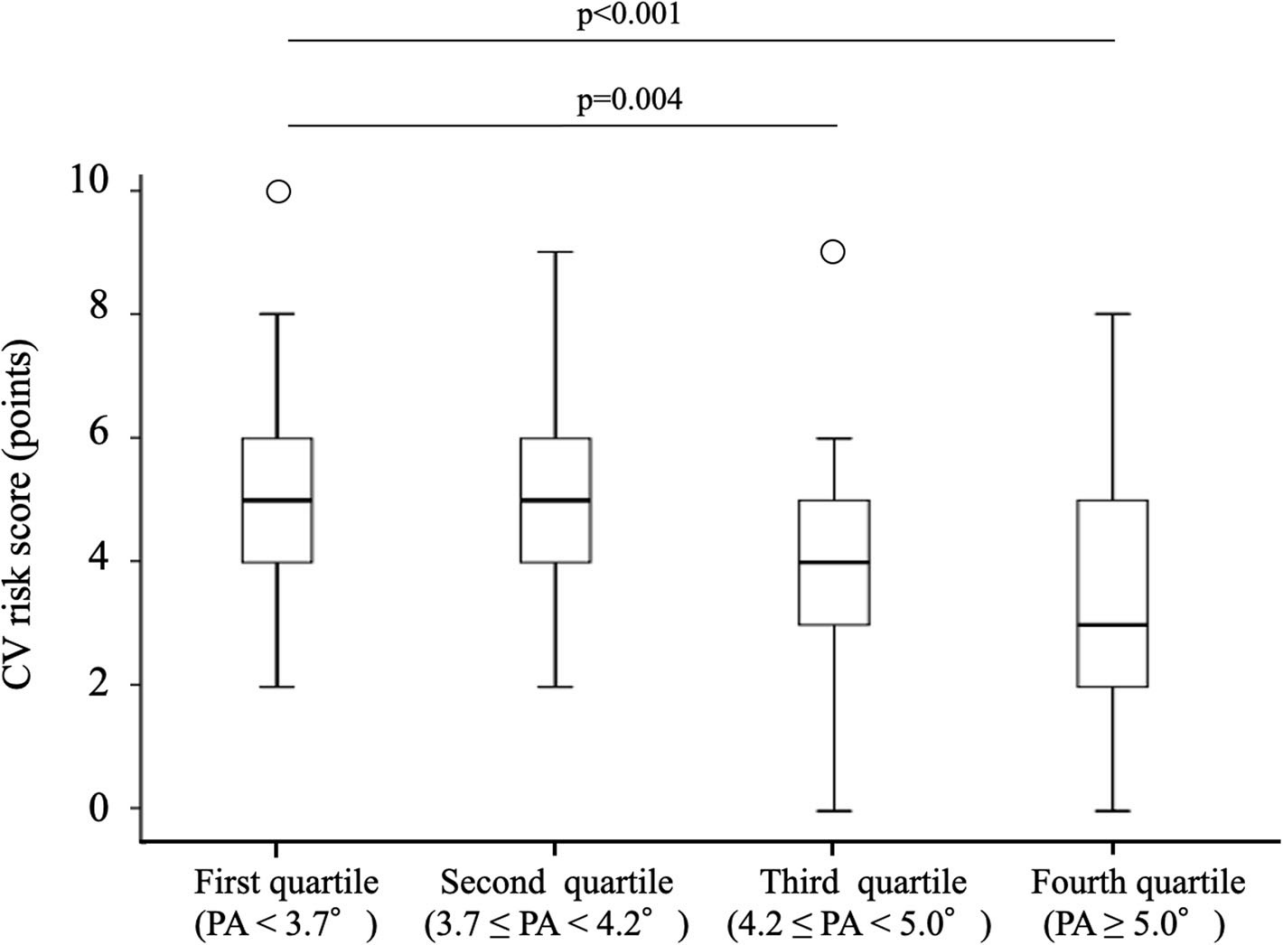
Phase angle and CV risk model score developed by J-DOPPS among HD patients. The box plots display the 50th (H), 25th and 75th (⊔⊓), 10th and 90th (−) percentiles and circles for the <10th and > 90th percentiles of the variable. CV, cardiovascular; PA, phase angle

Figure 1 shows the relationship between PA and four-quartile subgroups of CV risk model score among HD patients. The first quartile was significantly higher CV risk score compared with third and fourth quartile groups (*p* = 0.004 and *p* < 0.001).

## Discussion

PEW and frailty are common complication associated with functional decline, and worse prognosis in HD patients [7, 10, 11]. The present study demonstrated that a low PA measured by BIA as a simple alternative screening tool is an independent predictor of PEW and frailty as well as CV risk score in HD patients.

The biological meaning of the PA remains uncertain; however, it seems to reflect body cell mass, or cell membrane function [5]. Increased ECW is associated with poor nutritional status, and reduced total body water (TBW), an indicator of lower body cellular mass. Therefore, an increase in the ECW/TBW may be explained by malnutrition or skeletal muscle mass loss, as well as by fluid overload status [12]. Our findings also demonstrated that lower PA tended to have a significantly higher ECW/TBW ratio among HD patients. Moreover, a low PA showed a tendency for association with lower BMI, hand grip strength, and higher ECW-TBW and C-reactive protein levels, which are included in the PEW or frailty diagnostic criteria and were consistent with results from prior studies [13, 14]. Moreover, a lower PA correlated with PEW, the highest OR in the multivariate models was 10.967 and 11.099 in the first quartile group compared to the reference group (fourth quartile group). Ruperto et al. subsequently confirmed that PA < 4° was an independent risk predictor for HD patients with PEW [15], and is in line with our results. Thus, we also recognized that a low PA was an important indicator of malnutrition and hydration in HD patients. In addition, we showed that a low PA was associated with low TBW as an indicator of skeletal muscle mass in HD patients. Moreover, low PA was a greater risk factor for frailty even after adjusting for other clinical indicators. Several prior studies have reported an association between low PA and frailty phenomenon in older subjects [16, 17], or cardiac surgery patients [18], although very few studies have focused on HD patients [19]. In the present study, we also determined that low PA is a representative comprehensive biomarker of malnutrition, frailty, as well as hydration in HD patients.

Few studies have evaluated the association between PA and mortality or CV event in HD patients. More recently, Bansal et al. and Segall et al. demonstrated that PA was significantly associated with mortality in CKD patients [20] and HD patients [21]. Varan et al. reported a significant increase in the risk of death among HD patients with PA < 4°, even after adjustment of several nutritional indicators [22]. In present study, the first quartile group (PA < 3.7°) was at a significantly higher CV risk score compared with third (PA 4.2–4.9°) and fourth quartile (PA ≥ 5.0°) groups. However, given the relatively small number of patients and few events in present study, we could not perform analyses to identify factors related to all-cause mortality or incident of CV event.

Thus, we propose that regular screening would be essential to monitor the progression of PEW or frailty over time, and to avoid the development of the vicious cycle of PEW or frailty. Regular screening may help in the early identification of patients accompanied by PEW or frailty when they are the most treatable as well as provide prognostic information. We therefore suggest that PA could be a useful, simple indicator to predict PEW, frailty, and CV event risk among HD patients.

### Study limitations

Several limitations of our present study should be noted. First, our findings are limited to a relatively small number of patients at a single HD center, though most of the results are comparable to those from prior clinical studies. We have done sample size power calculation, a sample size of 122 patients was chosen based on the recommended method (median effect size 0.3, alpha err probability 0.05, power 0.8). Thus, our sample size is modest; further large number of studies are therefore needed in the future. Second, it has suggested that PEW is cachexia and should be termed kidney disease cachexia as a continuum with PEW first followed by cachexia. Our findings are limited to a small number of patients with severe PEW or kidney disease cachexia, therefore we could not assess the relationship between phase angle and severity of PEW including kidney disease cachexia. Future research needs to evaluate the diagnostic, prognostic, and predictive accuracy of phase angle on severe PEW or kidney disease cachexia status.

## Conclusion

PA could be a useful, simple indicator to predict PEW and frailty among HD patients. Lower PA was associated with a greater risk of PEW and frailty in hemodialysis patients.

## Data Availability

The datasets used and/or analyzed during the current study are available from the corresponding author on reasonable request.

## Abbreviations

BIA: Bioimpedance electrical analysis
BMI: Body mass index
BSA: Body surface area
CKD: Chronic kidney disease
CV: Cardiovascular
ECW: Extracellular water
HD: Hemodialysis
ISRNM: International Society of Renal Nutrition and Metabolism
J-CHS: Japanese version of Cardiovascular Health Study
J-DOPPS: Japan Dialysis Outcomes and Practice Patterns Study
nPNA: Normalized protein nitrogen appearance
ORs: Odds ratios
PA: Phase angle
PEW: Protein-energy wasting
SDS: Standard deviation score
TBW: Total body water

## References

[1] Kim JC, Kalantar-Zadeh K, Kopple JD (2013). Frailty and protein-energy wasting in elderly patients with end stage kidney disease. J Am Soc Nephrol.

[2] Johansen KL, Chertow GM, Jin C, Kutner NG (2007). Significance of frailty among dialysis patients. J Am Soc Nephrol.

[3] Worthen G, Tennankore K (2019). Frailty screening in chronic kidney disease: current perspectives. Int J Nephrol Renovasc Dis.

[4] Selberg O, Selberg D (2002). Norms and correlates of bioimpedance phase angle in healthy human subjects, hospitalized patients, and patients with liver cirrhosis. Eur J Appl Physiol.

[5] Garlini LM, Alves FD, Ceretta LB, Perry IS, Souza GC, Clausell NO (2019). Phase angle and mortality: a systematic review. Eur J Clin Nutr.

[6] Matsubara Y, Kimachi M, Fukuma S, Onishi Y, Fukuhara S (2017). Development of a new risk model for predicting cardiovascular events among hemodialysis patients: population-based hemodialysis patients from the Japan dialysis outcome and practice patterns study (J-DOPPS). PLoS One.

[7] Fouque D, Kalantar-Zadeh K, Kopple J, Cano N, Chauveau P, Cuppari L (2008). A proposed nomenclature and diagnostic criteria for protein-energy wasting in acute and chronic kidney disease. Kidney Int.

[8] Satake S, Shimada H, Yamada M, Kim H, Yoshida H, Gondo Y (2017). Prevalence of frailty among community-dwellers and outpatients in Japan as defined by the Japanese version of the cardiovascular health study criteria. Geriatr Gerontol Int.

[9] Bosy-Westphal A, Schautz B, Later W, Kehayias JJ, Gallagher D, Müller MJ (2013). What makes a BIA equation unique? Validity of eight-electrode multifrequency BIA to estimate body composition in a healthy adult population. Eur J Clin Nutr.

[10] Carrero JJ, Stenvinkel P, Cuppari L, Ikizler TA, Kalantar-Zadeh K, Kaysen G (2013). Etiology of the protein-energy wasting syndrome in chronic kidney disease: a consensus statement from the International Society of Renal Nutrition and Metabolism (ISRNM). J Ren Nutr.

[11] Obi Y, Qader H, Kovesdy CP, Kalantar-Zadeh K (2015). Latest consensus and update on protein-energy wasting in chronic kidney disease. Curr Opin Clin Nutr Metab Care.

[12] Woodrow G (2006). Extracellular water expansion: part of the malnutrition-inflammation-atherosclerosis syndrome?. Perit Dial Int.

[13] Beberashvili I, Azar A, Sinuani I, Shapiro G, Feldman L, Stav K (2014). Bioimpedance phase angle predicts muscle function, quality of life and clinical outcome in maintenance hemodialysis patients. Eur J Clin Nutr.

[14] Tan RS, Liang DH, Liu Y, Zhong XS, Zhang DS, Ma J (2019). Bioelectrical impedance analysis–derived phase angle predicts protein–energy wasting in maintenance hemodialysis patients. J Ren Nutr.

[15] Ruperto M, Sánchez-Muniz FJ, Barril G (2016). Predictors of protein-energy wasting in haemodialysis patients: a cross-sectional study. J Hum Nutr Diet.

[16] Wilhelm-Leen ER, Hall YN, Horwitz RI, Chertow GM (2014). Phase angle, frailty and mortality in older adults. J Gen Intern Med.

[17] Zanforlini BM, Trevisan C, Bertocco A, Piovesan F, Dianin M, Mazzochin M (2019). Phase angle and metabolic equivalents as predictors of frailty transitions in advanced age. Exp Gerontol.

[18] Mullie L, Obrand A, Bendayan M, Trnkus OMC, Moss E (2018). Phase angle as a biomarker for frailty and postoperative mortality: the BICS study. J Am Heart Assoc.

[19] Delgado C, Doyle JW, Johansen KL (2013). Association of frailty with body composition among patients on hemodialysis. J Ren Nutr.

[20] Bansal N, Zelnick LR, Himmelfarb J, Chertow GM (2018). Bioelectrical impedance analysis measures and clinical outcomes in CKD. Am J Kidney Dis.

[21] Segall L, Moscalu M, Hogaş S, Mititiuc I, Nistor I, Veisa G (2014). Protein-energy wasting, as well as overweight and obesity, is a long-term risk factor for mortality in chronic hemodialysis patients. Int Urol Nephrol.

[22] Varan HD, Bolayir B, Kara O, Arik G, Kizilarslanoglu MC, Kilic MK (2016). Phase angle assessment by bioelectrical impedance analysis and its predictive value for malnutrition risk in hospitalized geriatric patients. Aging Clin Exp Res.

